# Aerosol boxes for airway management in coronavirus disease patients: a clinical retrospective study in Mexico

**DOI:** 10.1186/s44158-022-00061-8

**Published:** 2022-07-19

**Authors:** Gilberto Duarte-Medrano, Massimiliano Sorbello, Rafael Renato Susunaga-Hope, Paula Ivette Fuentes-Castro, Beatriz C. Avila-OrtIz, Aida Paola Velasco-Godinez, Wendy Y. Contreras-Garcia, Miguel Alejandro Pineda-Castillo, Felipe Urdaneta, Félix Ignacio Téllez-Ávila

**Affiliations:** 1 Anesthesiology Department of the South-Central Hospital of High Specialty Pemex, Mexico City, Mexico; 2 Anaesthesia, and Intensive Care, Policlinico San Marco University Hospital, Viale C. A. Ciampi, 95100 Catania, Italy; 3 Clinical Professor Anesthesiology, Department of Anesthesiology, University of Florida/NFSGVHS, Florida, USA; 4 Gastrointestinal Endoscopy Department of the National Institute of Medical Sciences and Nutrition Salvador Zubirán, Mexico City, Mexico

**Keywords:** Aerosol box, Aerosol generating procedures, COVID-19, Droplets, Intubation

## Abstract

**Introduction:**

Significant concerns raise for the healthcare workers involved in airway management of patients diagnosed with coronavirus 2019 disease (COVID-19).

Due to shortages of personal protective equipment (PPE), barrier enclosure systems such as aerosol box (AB) have been proposed worldwide. The aim of this study was to evaluate our experience using AB as protective equipment in patients with COVID-19 in a third-level center in Mexico.

**Methods:**

A retrospective study of COVID-19 patients requiring airway management using an AB in the Hospital Central Sur de Alta Especialidad de Pemex in Mexico City from March 1 to June 1, 2020. Antropometric data, pre-intubation vital signs, and laboratory tests were recorded; the primary endpoints were intubation success rate and complications associated with AB and patients’ mortality. As a secondary endpoint, AB subjective evaluation was explored by administering a survey after airway management procedures.

**Results:**

Thirty-nine patients for a total of 40 intubations were documented. Thirty-one (77.5%) were men, with a mean age of 61.65 years; successful intubation occurred in 39 (97.55%) of the procedures, and AB was used in 36 (90%) of intubations, with success in 28 (70.0%); A Cormack-Lehane grade 3 view was recorded in 18 patients (46.2%), and during the procedure, the AB had to be removed in 8 (22.2%) cases, with migration documented in 91.6% of cases. The 30-day mortality was 48.71%, with 23.0% of patients discharged. 83.3% of surveyed anesthesiologists reported significant limitations in manipulating airway devices with AB used.

**Conclusion:**

Our data indicate that in clinical practice, the use of AB may hinder airway management and decrease the intubation success rate and may also result in patients’ injury. Further studies are necessary to validate the use of AB in clinical practice, and they should not replace certified PPE.

**Supplementary Information:**

The online version contains supplementary material available at 10.1186/s44158-022-00061-8.

## Introduction

The recent severe acute respiratory syndrome due to the novel coronavirus 2 (SARS-CoV2) posed a series of challenges for any healthcare system globally, including the capability to handle critically ill patients’ surge, triage and ethics concerns, the need to redesign logistics, clinical pathways, and protocols, and the need to deal with healthcare workers’ (HCWs) risk of infection and need of personal protective equipment (PPE) [1].

As of August 31, 2020, the impressive number of 25,721,294 confirmed cases and 856,289 deaths from coronavirus disease 19 (COVID-19) had been reported in the world, Mexico being the eighth country with the highest number of cases (599,560) and deaths (64,414), fifth in the American continent, after USA, Brazil, Peru, and Colombia [2].

SARS-CoV-2 may infect the host by either large droplets and aerosol particles, therefore require airborne-level PPE for HCWs, particularly during aerosol-generating procedures (AGPs) [3, 4].

As reported from other countries, HCWs have been affected by the COVID-19 infection, with around 12% of total infections in Italy [5], more than 150,000 calculated in Europe [6], more than 300 deaths in the USA by the end of May 2020 [7], and 978 confirmed deaths in Mexico by July 24, 2020 [8].

Other than correct social behavior and personal hygiene, specific protection policies for HCWs must include adequate PPE [9].

The overwhelming nature of the COVID-19 pandemic, the worldwide unpreparedness, and conflicting information with regard to protection indications [10, 11] have led to a global shortage of PPE and contributed to unavoidable risk overexposure for many HCWs [12], urgently requiring optimization policies and PPE prioritization [13]. As a response to the global shortage, after the initial idea of a Taiwanese doctor [14], the use of barrier enclosure systems was proposed as alternative methods to protect HCW’s during AGP [15, 16].

This study was designed to assess the clinical performance and to report the clinical experience using an AB for airway management of COVID-19 patients in a tertiary-level medical center in Mexico.

## Methods

A retrospective review was performed, accessing data from an electronic database of patients diagnosed with COVID-19 and requiring airway management in the Central Sur Hospital of High Specialty of Pemex, Mexico City, from March 1 to June 1, 2020.

Accessing the database, only patient with confirmed COVID-19-positive polymerase chain reaction (PCR) test were enrolled for subsequent analysis. Demographic data was collected (gender, age, weight, height, body mass index), past medical history (diabetes mellitus, high blood pressure, chronic kidney failure), and drug addiction (smoking, alcoholism). Vital signs (blood pressure, heart rate, temperature, respiratory rate, oxygen saturation), arterial blood gas (pH, lactate, HCO_3_), and laboratory data (platelets count, hemoglobin, hematocrit, leukocytes, neutrophils, glucose, creatinine) were collected before airway management.

Data regarding tracheal intubation were also collected (intubation date, use of an AB, drugs used, laryngoscope blade, use of introducer/bougie, endotracheal tube size, number of attempts, complications, evidence of AB displacement, or need for its removal). Whenever possible, data for documented SARS-CoV-2 infection of HCWs involved in airway management was also retrieved.

Primary endpoints of the study were intubation success rate, complications associated with AB, and patients’ mortality. As a secondary endpoint, the subjective evaluation of aerosol boxes was assessed with an ad hoc designed survey administered after airway management procedures to all anesthesiologists involved in the airway team any time the AB was used.

Inclusion criteria were confirmed positivity for COVID-19 as from a positive PCR with a nasal swab in patients requiring airway management, using an AB.

Exclusion criteria included lack of consent, patients intubated by other services besides anesthesia, missing data, and patients with known difficult airways.

Written informed consent was obtained from all patients, relative, or caregivers upon hospital admittance, and the study received ethical committee approval by the ethics committee of the South-Central Hospital of High Specialty with number 51-20.

The following definitions were used throughout the study:

- Positive patient: a patient diagnosed with COVID-19 with a positive PCR;
- Successful laryngoscopy: intubation achieved on the first attempt;
- Failed laryngoscopy/intubation: inability to visualize laryngeal structures and/or to place the tube into the trachea;
- AB migration: movement of the AB from the original position during airway instrumentation (original position was marked with tape);

Operator discomfort for intubation: any limitation for natural movements because of the AB ports and/or perceived limitation for airway instrumentation.

**Fig. 1:**
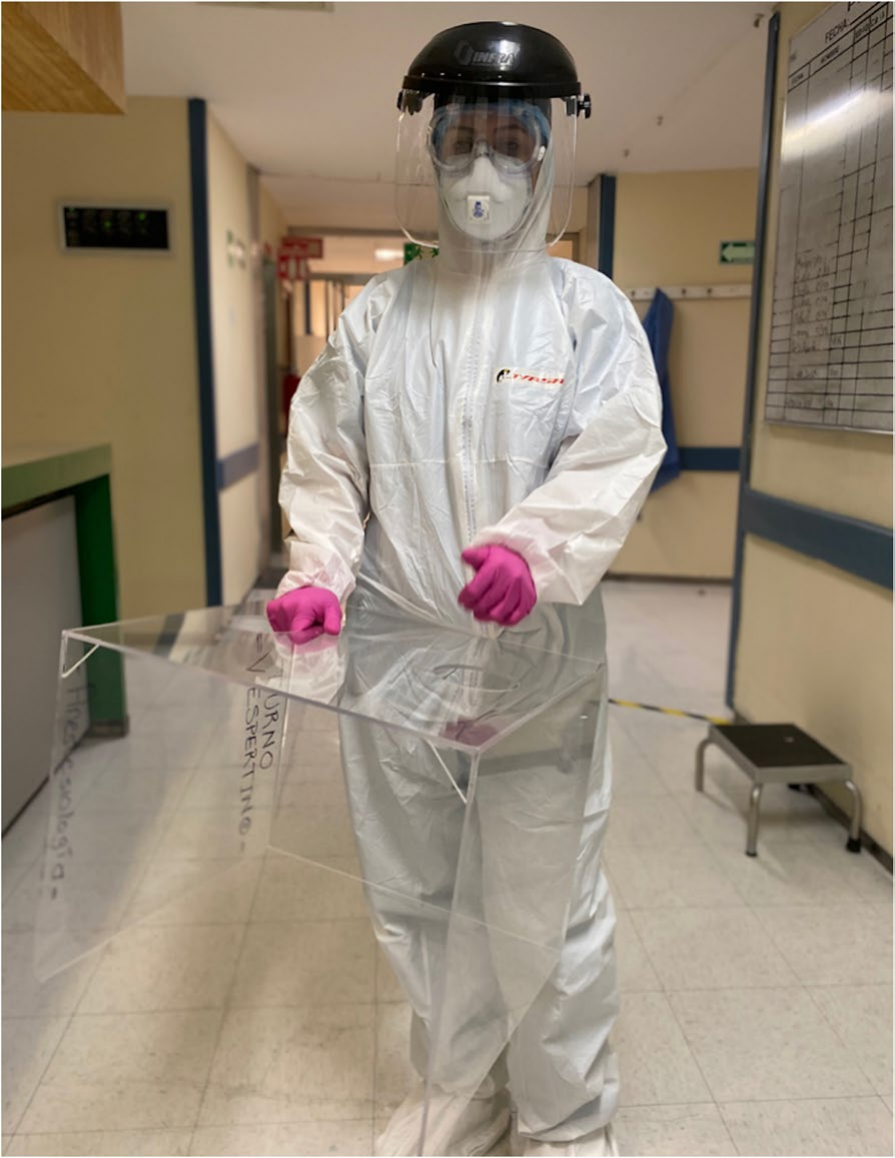
Anesthesiology with personal protective equipment and aerosol-box prior to intubation of a COVID-19 patient

All intubations were performed by a two-anesthesiologists airway team, both of them senior anesthesiologists with residents only occasionally being included in the team and not with primary role. As suggested from different COVID-19 airway guidelines [5, 9, 13], the most experienced was performing laryngoscopy and intubation, and the second one assisted and administered rapid sequence induction/intubation (RSII) medications being ready to take the lead in case of failure of the first intubator. Given the pandemics scenario, the use of airway boxes was relatively new to any team member, whereas all team members used PPE following the national guidelines for the intubation of a patient with COVID-19: N95 respirator plus a surgical mask, goggles, water-resistant gown, and double gloves (Fig. 1).

The intubation device’s choice was left to the airway team leader, with two options for a standard Macintosh laryngoscope (with available blades sizes 3 and 4) or a King Vision video laryngoscope (AMBU, Ballerup, Denmark) with a size 3 blade. Adjuncts for laryngoscopy included malleable stylet or tracheal introducer.

**Fig. 2:**
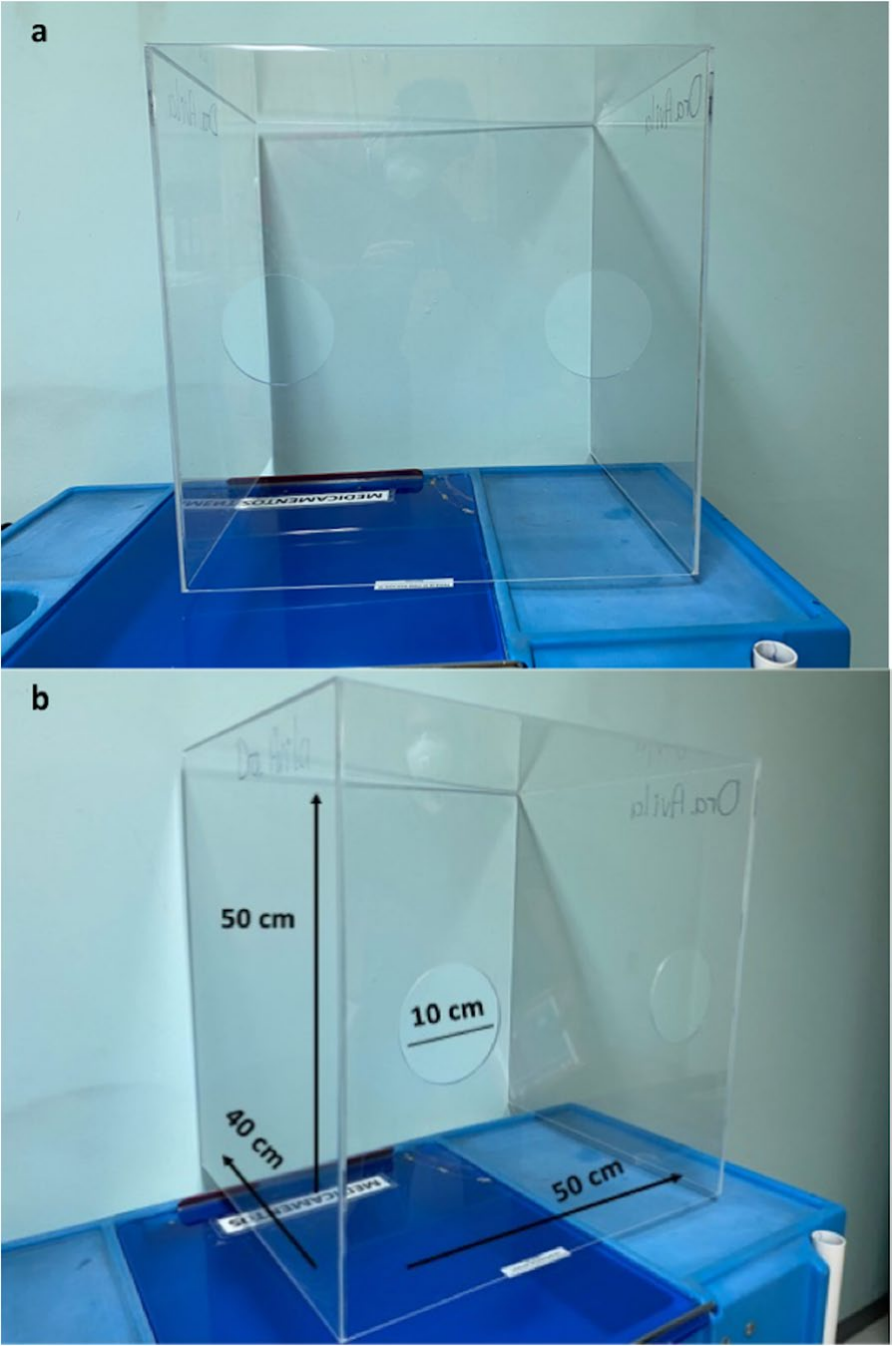
**A**, **B** Dimensions of aerosol-box used as barrier protection in the approach to the airway in the Hospital Central Sur de Alta Especialidad, Mexico

The AB used for airway management was a “first-generation” box consisting of a transparent acrylic cube, 50 cm (20 in) high x 50 cm (20 in) wide x 40 cm (16 in) deep, with two 10 cm (4 in) circular openings at 25 cm (10 in) from the base and 5 cm from the lateral face on the operator’s side (Fig. 2a, b).

**Fig. 3:**
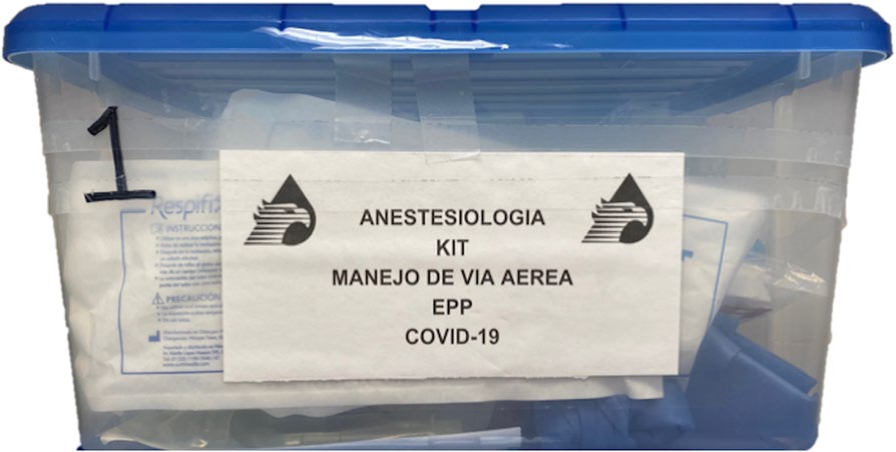
The COVID-19 dedicated airway box used for airway management and placed close to patient together with the aerosol box

Airway evaluation was briefly performed before initiating the airway instrumentation procedure; this approach was maintained also in case of emergency intubations, taking account that expert anesthesiologists were always included in the airway team. Strategies to optimize peri-procedural oxygenation were undertaken during airway evaluation and patient’s preparation. The intubation procedure was performed after positioning the patient, and once the AB was placed above the patient’s head, with adhesive tape used to mark AB position and to secure it. A dedicated airway box was prompted close to the patient (Fig. 3) and after 3–5 min of preoxygenation performed with a Waters circuit with double filters setting in 100% oxygen, RSII was performed with fentanyl (3–4 mcg*kg^-1^), propofol (1–2 mg*kg^-1^), and rocuronium (1 mg*kg^-1^). Intubation attempted 60 s later. No manual ventilation was provided unless desaturation occurred and no cricoid pressure applied unless clear signs of regurgitation/aspiration.

The laryngoscope was introduced on the left orifice of the AB. The right orifice was used first for head manipulation, and the introduction of the endotracheal tube occluded with a Rochester clamp. After successful intubation, as confirmed by chest auscultation, peripheral saturation, and end-tidal carbon dioxide (when available), the operator’s arms were removed from the ports of the AB, the laryngoscope was placed in a bag for disinfection, the AB was removed after observation of original position landmarks, and the patient was transferred to the intensive care unit (ICU).

In the event of an intubation failure, a second attempt was performed by the same or the second team member; after two failed attempts, or upon decision of the airway team leader, the AB was removed assuming it may cause interference, limitation, or potential harm.

After use, the AB was cleaned and sanitized with a 10% sodium hypochlorite solution. After the procedure, the airway team members completed a nine items satisfaction survey for the AB use evaluating protection, comfort, problems with eventual migration, and the possibility for assistance by the second operator.

A further question was also addressed, investigating any proposal of possible modifications and changes in the AB, and the final question was whether they would recommend its use. The survey was administered in electronic format and was entirely anonymous, and it was prepared ad hoc for the study given the lack of studies for in vivo clinical use of AB for COVID-19 patients airway management. The survey text is available in Additional file 1.

### Statistical analysis

The results were evaluated using descriptive statistics for the non-parametric distribution: means, standard deviation and minimum-maximum intervals, and absolute and relative frequencies. The analysis was performed with the statistical package IBM SPSS Statistics 24.0 for Mac (IBM, New York, USA).

## Results

A total of 40 intubations in 39 patients were performed (one patient was intubated twice). Thirty-one patients (77.5%) were men, with a mean age of 61.65. Patient information and comorbidities are listed in Table 1; 17 (42.5%) of cases showed grade 1 obesity, and diabetes mellitus was present in 23 (57.3%) cases.

**Table 1:** COVID-19 patients’ characteristics

Patients’ sample characteristics	***n*** (%)
Male/female	30/9 (76.9/23.1)
**Nutritional condition**	
Normal weight	5 (12.8)
Overweight	10 (25.6)
Obesity grade 1	17 (43.6)
Obesity grade 2	6 (15.4)
Obesity grade 3	1 (2.6)
**Chronic degenerative diseases**	
Diabetes mellitus type 2	22 (56.4)
Arterial hypertension	19 (48.7)
Cancer history	9 (23.1)
Smoking	5 (12.8)
**COVID-19 symptoms**	
Dyspnoea	33 (84.6)
Cough	29 (74.4)
Fever	16 (41)
Odynophagia	25 (64.1)
Headache	13 (33.1)
Myalgia	6 (15.4)
Arthralgia	5 (12.8)
Diarrhea	5 (12.8)
**Anthropometrics**	**mean (range)**
Age (years)	62.4 (31-88)
Weight (kg)	82.4 (50-117)
Height (m)	1.64 (1.47-1.78)
Body mass index	30.39 (22.2-40.50)

The most common symptom requiring intubation team activation and evaluation for intubation was dyspnea in 34 cases (85%), with a mean oxygen saturation before intubation of 79.8%.

Overall, success with tracheal intubation was achieved in 39 (97.55%) of cases. The AB was used for 36 (90%) intubations. In the remaining 4 cases (10%), the AB use was excluded before the procedure because of the operator’s discomfort in 3 cases and because of the patient’s size in 1 case.

In all cases, when the AB was used, the first attempt was successful in 28 cases (70.0%). In 8 patients (22.2%), it had to be removed because of failed intubation after multiple attempts with evidence of oropharyngeal injury (two attempts were considered as decisional trigger to remove the AB or as decision of the airway team leader). Given the paucity of available videolaryngoscopes in Mexico during pandemic and in lack of experience with their use, a standard Macintosh laryngoscope was used in all procedures, using a number 3 blade in 37 cases (94.9%) with an endotracheal tube 8 mm (20 cases), 7.5 mm (14 cases), and 7 mm (5 cases) inner diameter, and a tracheal introducer was used in 5 patients (12.8%).

Mortality was followed-up at 30 days, with 19 fatalities (48.71%), 9 (23.0%) discharges, and 12 (30.76%) patients still in intensive care at the time of writing this paper.

Twelve anesthesiologists performed the 40 procedures and completed the post-procedural survey.

Ten anesthesiologists (83.33%) reported they felt safe using the AB, despite 11 (91.66%) reported a certain discomfort during the first uses of the AB. Eight (66.66%) claimed significant limitations in manipulating airway devices, with 10 (83.33%) reporting contact with the box walls. Migration of the AB was reported in 11 cases (91.66%), and in 10 patients (83.33%), help was required for the mobilization of the AB during the procedure.

Between the proposed changes in the design of the AB, 11 anesthesiologists (91.66%) suggested to reduce height, 7 (58.33%) proposed a width reduction, and 4 (33.33%) larger ports. Overall, only 9 (75%) anesthesiologists would recommend AB for the intubation of COVID-19 patients.

Among the anesthesiologists involved in the airway teams, 3 of them (25%) had documented SARS-CoV-2-positive nasal swabs at the end of the study.

## Discussion

Our study shows that use of AB impedes airway management, with risks for patients in terms of intubation outcome and in terms of potential harm; as a further remark, the use of AB seems to superimpose infection risks for HCWs with evidence of increased cognitive and physical workload.

COVID-19 represents an unprecedented challenge for healthcare system organization and facilities [17] with significant efforts and tasks for HCWs exposed to high-infection risk given the nature of the pathogen agent [18–20]. Data from the international and multicentric intubateCOVID-study indicate that 1 in 10 HCWs taking care of airway management develops SARS-CoV-2 infection [21], not underestimating physical and mental health issues due to increased workload [22]. Implications of such phenomena on endurance, performance, and safety of airway management teams are of paramount importance, either during a pandemic event and during endemic/silent phases, given the potential long-term effects in terms of fatigue, burnout, and risk of death [23, 24]. The use of PPE and of AB is a further load to be considered, especially during airway management, and to our knowledge, this is one of the first reports of clinical data on the performance and impact of barrier enclosure systems on airway management.

A recent review [16] suggests that barrier enclosures may impede airway management, limit hands-on time if help is needed, and may complicate the use of some airway devices, such as tracheal introducers [25] or advanced airway manipulation as for one-lung ventilation procedures [26]. Simulation studies suggest longer intubation times with barrier enclosure systems, with a first pass success rate of 75% when using first-generation AB [27], and these data were supported also by a recent systematic review [28].

Our data indicate that when the AB was used (90% of intubations), the first-pass intubation success rate was 70%. In 22.2% of cases, and the AB was removed because of multiple intubation attempts, with documented traumatic complications during airway management. This observation is in line with experimental studies and experts’ hypothesis as from relevant literature [16], supporting the evidence that use of AB has a significant interference with ergonomics of airway management, and it may add extra cognitive and operational tasks to operators which are already under heavy psychological and physical pressure. We did not record the intubation time, but our data suggest that airway management was prolonged and complicated by the use of AB in many cases, including a reported incidence of 46.2% for Cormack-Lehane grade 3 laryngeal view; of notice, given the limited availability and lack of experience, a videolaryngoscope was not used for any intubation.

This finding may represent a bias, given that acquisition of line of sight with standard laryngoscope may be hindered by the optical and physical interface represented by the AB wall. Also, the need for intubation out of operatory room but in medical wards may have added difficulty to airway management, and this could be considered a biasing factor in AB evaluation.

As a subjective data, 66.6% of physicians participating the study reported significant airway management limitations.

Our study also raises concerns for patients and HCWs’ safety: the AB migrated during airway management in 91.66% of cases, which may have multiple implications: first, the box movement may complicate or compromise airway management attempts. Secondly, should the box move or fall from the bed, it may hit hurt the patient or the attending physician; last but not least, the box dislodgment may compromise the infection-control principle, if any.

Interestingly, 83.3% of the providers reported physical contact with the box walls, with 33.3% suggesting the use of larger access ports. As reported in Begley’s simulation study [27], this contact may also result in PPE damage with increased infection risk.

Perception of increased difficulty in airway management and risks for patients and HCWs was a common concern: In 10% of cases, the intubation team decided not to use the AB prior to the procedure either for comfort or patient’s size or perceived risk of difficult intubation during pre-procedural evaluation.

The AB’s perceived protection was somewhat controversial: 83.3% of surveyed providers reported feeling safe with the device, whereas in 92% of cases some kind of discomfort was reported, and only 75% of participants recommended the use of barrier enclosure systems when filling the post-procedural report.

Our study was underpowered to detect infection risks or incidence in HCWs, and it was not designed for such an endpoint. Nevertheless, at the end of the study, 25% of the intubation team members had a SARS-CoV-2-positive PCR nasal swab test, verisimilarly related to a clinical exposure.

We may not exclude that an infection route was some aerosolization associated with the use of AB. As elegantly demonstrated in recent simulation studies, these devices may effectively stop the large droplets but they may not stop aerosols, with particles escaping through access ports via Bernoulli effect [29] or upon removal (“secondary aerosolization”) [16, 30].

Concerns about safety and performance, and overall effects on airway management success, prompted the FDA to revoke the permit of AB in August 2020, so that barrier enclosures without the possibility of additional negative pressure are no longer recommended [31].

Recent studies also highlight that any not fully enclosed AB may redirect aerosol toward the laryngoscopist, indicating that only an aerosol evacuation system may reduce aerosols inside the box, which should never substitute, but complementary to certified PPE [32, 33].

Interestingly, recent evidence suggests that extubation may be even more critical in terms of aerosol generation and consequential infective risks [34]; the use of AB or barrier enclosures may be even more challenging during extubation [16, 35], with poorly cooperative if not combative patients and with few medications to counteract extubation associated phenomena (coughing, nausea, vomiting, etc.). Operator’s discomfort, the risk of AB damage or patients’ injury was also largely perceived during extubation phase, and in all cases when AB was removed or not used during initial airway management, it was not used a priori for extubation.

With 2.5% of all COVID-19-diagnosed Mexican patients requiring endotracheal intubation and invasive mechanical ventilation and a global 73.7% mortality [36], Mexico was heavily hit by the pandemic [37], paying the price of one of the highest levels of infected HCWs globally.

46,013 (22.08%) of young (30––54 years old) Mexican HCWs developed a SARS-CoV-2 infection. The majority of them were not hospitalized, but between the 693 in critical condition, 234 required endotracheal intubation, with a case fatality rate of 1.48% (683/46 013) within HCWs compared with a global 15.26% [38, 39]. A report from the Amnesty International indicated that Mexico had the world highest number of deaths between HCWs (1320), followed by USA (1077 deaths) and UK (677 deaths) [40].

PPE per se do not protect, unless users are not well trained in their use, in donning and doffing procedures and only if PPE are part of a wider strategy and preparedness [41], including protocols, logistics, and availability of well trained and specialized physicians [17, 36] with an educated and consolidated teamwork approach [42]. The use of barrier enclosure systems in countries like Mexico may reflect the need to achieve some means of protection when conventional and certified PPEs are lacking [43]. Nevertheless, our clinical data confirm that barrier enclosure systems add complexity to airway management; they may represent a risk for patients’ safety, they may expose HCWs to a higher risk of infection when used as surrogates or substitutes of conventional PPE, and they may finally provide a false sense of safety thus resulting in lower attention and perception of lower personal protection needed, not considering the risk of PPE damage because of contact or impingement with the AB.

Our study has limitations as follows: first, it is a retrospective study conducted during the pandemic surge, and some data may have been missed or misreported. The survey responses were subjective, but we believe they may reflect quite objectively the clinical reality and HCWs feelings.

We did not collect intubation duration data, nor could we carefully analyze data of HCWs infection rates, because of the study’s design, with a control arm missing.

On the other hand, to our knowledge, this is one of the first clinical studies evaluating the use of AB in airway management in a series of 40 COVID-19 patients.

## Conclusions

Based on our experience, the use of barrier enclosure systems for HCW protection during COVID-19 airway management added complexity to airway instrumentation in our patients’ cohort. The use of AB did not seem to reduce the risk of HCW infection while increasing cognitive and physical loads, and the majority of our providers did not recommend the use of such devices. Proper use and adequate training in the use of PPE and development of training and organizational programs, including availability of medical equipment and social practices, should remain the primary options and gold standards for the protection of HCW’s during AGPs.

Further studies are necessary before proposing the clinical use of AB and barrier enclosure systems.

## Supplementary Information

**Additional file 1.** Aerosol-box questionnaire in COVID-19 patients.

## Data Availability

Address any request to Gilberto Duarte-Medrano—Anesthesiology Department of the South-Central Hospital of High Specialty Pemex, Mexico City, Mexico.
