# Supplementary material for: Aerosol boxes for airway management in coronavirus disease patients: a clinical retrospective study in Mexico
Source: J Anesth Analg Crit Care. 2022 Jul 19;2:32. doi: 10.1186/s44158-022-00061-8 (PMC10246106; doi:10.1186/s44158-022-00061-8)
Supplement: Supplementary file 1 — Additional file 1. Aerosol-box questionnaire in COVID-19 patients. [file 44158_2022_61_MOESM1_ESM.docx]

Appendix 1

**AEROSOL-BOX QUESTIONNAIRE IN COVID19 PATIENTS**

**The following questionnaire is directed to the anesthesiologist in charge of airway management of COVID-19 patients usingf the “Aerosol-box” protection device.**

Please circulate or mark in the corresponding box:

1. Do you feel protected with the use of the Aerosol-Box?

Yes

No

No

1. Did you feel uncomfortable when using the Aerosol-Box for the first time?

Yes

No

1. Do you think that the use of the Aerosol-Box limits the handling of airway devices (King Vision, bougie, supraglottic devices?)

Yes

No

Yes

1. Did the laryngoscope you used contact the Aerosol-Box and limited its use?

Yes

No

1. Did you have box migration on the bed during intubation?

Yes

No

1. Did you require the help of the second anesthesiologist to hold the box?

No

Yes

1. Would you prefer not to have used the spray-box?
2. From the following options, check which aspects you would modify

*Box height*

*Box width*

*Hand ports dimensions*

Yes

No

1. Would you recommend the use of the Aerosol-Box?
